# Oral nano‐curcumin formulation efficacy in the management of mild to moderate outpatient COVID‐19: A randomized triple‐blind placebo‐controlled clinical trial

**DOI:** 10.1002/fsn3.2226

**Published:** 2021-06-19

**Authors:** Reza Ahmadi, Soofia Salari, Mohammad Davood Sharifi, Hamidreza Reihani, Mohammad Bagher Rostamiani, Morteza Behmadi, Zhila Taherzadeh, Saeed Eslami, Seyed Mahdi Rezayat, Mahmoud Reza Jaafari, Sepideh Elyasi

**Affiliations:** ^1^ Department of Family Medicine School of Medicine Mashhad University of Medical Sciences Mashhad Iran; ^2^ Department of Clinical Pharmacy School of Pharmacy Mashhad University of Medical Sciences Mashhad Iran; ^3^ Faculty of Medicine Department of Emergency Medicine Mashhad University of Medical sciences Mashhad Iran; ^4^ Department of Internal Medicine Mashhad University of Medical Sciences Mashhad Iran; ^5^ Targeted Drug Delivery Research Center School of Pharmacy Mashhad University of Medical Sciences Mashhad Iran; ^6^ Faculty of Medicine Department of Medical Informatics Mashhad University of Medical Sciences Mashhad Iran; ^7^ Department of Pharmacology School of Medicine Tehran University of Medical Sciences Tehran Iran; ^8^ Nanotechnology Research Center Pharmaceutical Technology Institute Mashhad University of Medical Sciences Mashhad Iran; ^9^ Department of Pharmaceutical Nanotechnology School of Pharmacy Mashhad University of Medical Sciences Mashhad Iran

**Keywords:** anti‐inflammatory, clinical response, COVID‐19, Curcumin

## Abstract

**Background:**

Curcumin, a natural polyphenolic compound, is proposed as a potential treatment option for patients with coronavirus disease by inhibiting the entry of virus to the cell, encapsulation of the virus and viral protease, as well as modulating various cellular signaling pathways. In this study, the efficacy and safety of nanocurcumin oral formulation has been evaluated in patients with mild‐moderate Coronavirus disease 2019 (COVID‐19) in outpatient setting.

**Methods:**

In this triple‐blind randomized placebo‐controlled clinical trial, sixty mild to moderate COVID‐19 patients in outpatient setting who fulfilled the inclusion criteria were randomly allocated to treatment (*n* = 30) group to receive oral nanocurcumin formulation (Sinacurcumin soft gel which contains 40 mg curcuminoids as nanomicelles), two soft gels twice a day after food for 2 weeks or placebo (*n* = 30) group. Patients’ symptoms and laboratory data were assessed at baseline and during follow‐up period and compared between two groups.

**Results:**

All symptoms except sore throat resolved faster in the treatment group and the difference was significant for chills, cough and smell and taste disturbances. The CRP serum level was lower in the treatment group at the end of two weeks and the lymphocyte count was significantly higher in treatment group. No significant adverse reaction reported in the treatment group.

**Conclusion:**

Oral nanoformulation of curcumin can significantly improve recovery time in patients with mild to moderate COVID‐19 in outpatient setting. Further studies with larger sample size are recommended.

## Graphical abstract

In this triple‐blind randomized placebo‐controlled clinical trial, sixty mild to moderate patients in outpatient setting were randomly allocated to nanocurcumin (*n* = 30) group to receive oral nanocurcumin formulation (Sinacurcumin soft gel which contains 40 mg curcuminoids as nanomicelles), two soft gels twice a day after food for 2 weeks or placebo (*n* = 30) group. All symptoms except sore throat resolved faster in the treatment group and the difference was significant for chills, cough and smell and taste disturbances. The CRP serum level was lower in the treatment group at the end of 2 weeks and the lymphocyte count was significantly higher in treatment group. No significant adverse reaction reported in the treatment group.

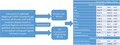

## 1 INTRODUCTION

Severe acute respiratory syndrome‐coronavirus‐2 (SARS‐CoV‐2) is first detected in December 2019 in China, and has been presented as the first pandemic of the century in March 2020. (Davoudi‐Monfared et al., 2020) The disease is named as coronavirus disease 2019 (COVID‐19) and dyspnea, fever, cough, myalgia and other flu‐like symptoms are its main presentation. (Grasselli et al., 2020) Though, it can progress to more severe disease and causes acute respiratory distress syndrome (ARDS), organ failure and death. (Mehta et al., 2020; Wu et al., 2020) It has caused more than 300,000 deaths worldwide in less than five months. (Rodriguez‐Morales et al., 2020) The attachment of SARS‐CoV‐2 to the toll‐like receptor results in the release of pro‐IL‐1β that is cleaved by caspase‐1, which activates inflammasome and generates active mature IL‐1β, mediating pulmonary inflammation and fibrosis. (Conti et al.,) Transforming growth factor (TGF‐ß) and its signaling pathways are also involved in lung fibrosis and its overexpression is associated with poorer prognosis in ARDS. (Scotton & Chambers, 2007) So, Cytokine profile (IL‐2, IL‐7, granulocyte colony stimulating factor, interferon (INF)‐γ inducible protein 10, monocyte chemoattractant protein (MCP) 1, macrophage inflammatory protein (MIP) 1‐α, and tumor necrosis factor (TNF)‐α) is a descriptive criteria for severity COVID‐19 disease. (Mehta et al., 2020; Richardson et al., 2020) However, until now supportive care measures and oxygen therapy remain the mainstay of COVID‐19 management and no approved effective treatment is available.

Curcumin is a derivative of *Curcuma longa* (turmeric), is a natural bioactive polyphenolic compound isolated from the dried powder turmeric rhizomes, and widely used worldwide as yellow spice. (Roy et al., 2020) Curcumin has various pharmacological effects such as antioxidant, anticancer, antibacterial, antiviral, antidiabetic and anti‐inflammatory activities. (Babaei et al., 2020) Curcumin can decrease excretion of various cytokines which have critical role in many severe and chronic disease [IL‐1, 2, 6, 8, 10, 11, 12, and 17, TNFα, interferon‐γ, MCP1, MIP1α, nuclear factor kappa‐light‐chain enhancer of activated B cells (NFĸB), cyclooxygenase (COX), plasminogen activator inhibitor‐1 (PAI‐1), and caspase‐3 (Cas‐3)]. (Babaei et al., 2020; Zahedipour et al., 2020).

Additionally, curcumin showed to have antiviral effects against a variety of viruses such as human immunodeficiency virus (HIV) −1, HIV2, herpes simplex virus (HSV), human papillomavirus (HPV), human T‐lymphotropic virus‐1 (HTLV1), hepatitis B virus (HBV), HCV, influenza A and Japanese encephalitis virus. (Zorofchian Moghadamtousi et al., 2014) Besides, curcumin 500 mg BD daily for 30 days in patients with bronchial asthma significantly improved forced expiratory volume one second (FEV1) in comparison with standard therapy. However, the mean scores for cough, dyspnea, wheezing, chest tightness, and nocturnal symptoms did not change significantly. (Babaei et al., 2020)

On the other hand, several clinical trials have revealed low oral bioavailability of curcumin. (Kunnumakkara et al., 2019) It is suggested that manipulation and encapsulation of curcumin into micelles, liposomes, phospholipid complexes, exosomes, or polymeric nanocarrier formulation and also utilization of curcumin in combination with cellulosic derivatives, natural antioxidants, and a hydrophilic carrier could increase its bioavailability. (Moballegh Nasery et al., 2020).

In a recent study in Iran, forty patients with COVID‐19 induced ARDS received nanocurcumin (Sinacurcumin soft gel 40 mg, 160 mg/d, for 14 d) or placebo randomly. After 14 d treatment with nanocurcumin, a significant decrease in IL‐6 and 1β expression and secretion in serum and in supernatant was observed. (Valizadeh et al., 2020).

In an open label clinical trial which was performed by our research team on mild‐moderate hospitalized COVID‐19 patients, forty‐one patients who fulfilled the inclusion criteria were allocated to nanocurcumin (*n* = 21) (Sinacurcumin soft gel 40 mg, four times a day, 160 mg/d) or control (*n* = 20) group for 2 weeks. Most of symptoms including fever and chills, tachypnea, myalgia, and cough resolved significantly faster in curcumin group. Moreover, (arterial oxygen saturation) SaO2 was significantly higher in treatment group after 2, 4, 7 and 14 days of follow‐up and lymphocyte count after 7 and 14 days. Length of need to supplemental O2 and hospitalization time was also meaningfully shorter in treatment group. It is also noteworthy to mention that no patient in treatment group experienced deterioration of infection during follow‐up period but it occurred in 40% of control group. (SaberMoghaddam et al., 2021

Besides, curcumin safety has been proven in several clinical trials. The allowable daily intake (ADI) value of curcumin has been reported 0–3 mg/kg and up to 12 g/day was tolerable. Diarrhea, headache, rash, and stool lightening were the most common reported adverse reactions with curcumin. (Akbari et al., 2020)

Meanwhile, the safety and efficacy of nanocurcumin (Sinacurcumin) also has been proven in different clinical trials. (Rahimi et al., 2016)^‐^(Ahmadi et al., 2018)

In this study, we evaluated the nanocurcumin oral formulation efficacy as an adjuvant for the management of mild to moderated outpatient COVID‐19 patients.

## 2 METHODS

### 2.1 St*udy design*

This study conducted as a randomized triple‐blind clinical trial from April to July 2020 at Edalatian emergency department, Imam Reza Hospital affiliated to Mashhad University of Medical Sciences, Mashhad, Iran.

### 2.2 Study population

Patients who fulfilled the following inclusion criteria are included to the study: Diagnosis of COVID‐19 based on (1) a positive real‐time polymerase chain reaction (RT‐PCR) of the respiratory tract samples, or (2) imaging findings highly suspicious for COVID‐19 (e.g., ground‐glass pattern in chest X ray) and clinical signs/symptoms, age between 18 and 65 years with mild to moderate disease based on national diagnosis and treatment guideline (last available version), who were to be treated in outpatient setting (no severe dyspnea, respiratory rate less than 30/min, SaO2 higher than 93% in room air) who signed informed consent. Patients did not include if more than 7 days passed from their onset of symptoms, be pregnant or breastfeeding,, smoking more than 5 cigarettes a day, had history of hypersensitivity to turmeric or curcumin compounds or concomitant disease including severe renal failure (eGFR < 30 ml/min), hepatic failure (Child‐Pugh Score B or C), heart failure (EF < 40%), chronic lung disease, active malignancy, auto‐immune disease, immune system impairment like HIV, gallbladder stone, and active gastrointestinal bleeding. Patients were excluded from study if they were hospitalized because of infection exacerbation, or severe drug adverse reactions occurred.

### 2.3 Ethics

The local Ethics Committee of Mashhad University of Medical Sciences approved the study protocol (Code: IR.MUMS.REC.1399.054), and it was registered at the Iranian Registry of Clinical Trials (IRCT20200408046990N1). All participants were explained and informed for protocol of study and signed written consent forms.

### 2.4 Study protocol

All included patients randomly placed in placebo or treatment (nanocurcumin) group. The herbal medicine used in this trial was Sinacurcumin® soft gel 40 mg, which is registered product from curcuminoids in Iran (IRC:1,228,225,765) and are industrialized in Nanotechnology Research Center of Mashhad University of Medical Sciences, marketed by Exir Nano Sina Company, Tehran, Iran. (Hatamipour et al., 2019) The treatment group received 2 soft gels after breakfast and 2 soft gels after dinner daily for 2 weeks. Placebo soft gels were prepared by the same company, in exactly the same appearance containing all ingredients of medicine soft gel except curcumin with same dosing (2 soft gel twice daily after meal). Patients in both groups received standard treatment based on national diagnosis and treatment guideline (last available version). Same nutritional recommendations were presented to all participants. Furthermore, they asked not to receive any other medications for COVID‐19 management without consultation with us. If infection exacerbation occurred, the patients were excluded from the trial and they were managed based on available guidelines.

### 2.5 Outcome

Patients demographic data and past medical and drug history were asked and recorded at the beginning of the study by the pharmacist. Moreover, patients were assessed by the emergency medicine specialist and pharmacist considering the various signs and symptoms of COVID‐19 infection (including fever, chills, cough, headache, sore throat, anosmia and taste disturbance, myalgia, weakness, dyspnea, gastrointestinal and dermatologic disorders) at the beginning of the study and daily thereafter by phone call, until complete resolution. Besides, laboratory data (CRP serum level and lymphocytes count), were recorded at baseline and after 2 weeks of treatment. The time of each symptom resolution and the CRP serum level and lymphocytes count changes during follow‐up period were considered as primary outcome of the study. COVID‐19 rt‐PCR and/or lung CT scan if available were also recorded at baseline. Moreover, we considered patients in deterioration situation if their symptoms did not resolve or exacerbate in 2‐week follow‐up period, their laboratory findings did not improve, or worsened or the SaO2 reduced in a manner that supplemental O2 became necessary.

During the study period, patients were also followed for compliance to treatment and adverse drug reactions as secondary outcome. They were proposed to be adherent to their treatment if they consumed more than 80% of their administered medicine/placebo. (Ho et al., 2009).

### 2.6 Sample size

As to the best of our knowledge this was the first clinical study on curcumin oral formulation efficacy for management of mild to moderate COVID‐19 infection, we proposed it as a pilot study and defined the sample size 30 patients in each group. Based on Whitehead et al. recommendation (Whitehead et al., 2016), for a main trial designed with 90% power and two‐sided 5% significance, pilot trial sample sizes per treatment arm of 75, 25, 15 and 10 is enough for standardized effect sizes that are extra small (≤0.1), small (0.2), medium (0.5) or large (0.8), respectively. So proposing the curcumin effect even small, 30 patients in each arm could be acceptable.

### 2.7 Randomization and blinding

The random allocation sequence was generated using a computer‐generated randomized list (accessed from randomization.com site). Placebo and nanocurcumin were filled in bottles and numbered (a number between1 and 60) based on the allocation sequence, by Exir Nano Sina Company. The boxes were delivered to the patients who fulfilled the inclusion criteria respectively by the pharmacist or the emergency medicine specialist. Each box contained 56 soft gels which was enough for 14 days. Assessment of patients during the treatment course was performed by the pharmacist. Data collection and insertion in SPSS software were carried out by the pharmacist. Before analysis of data by the clinical pharmacist, grouping data were added to the SPSS file by a person from the Exir Nano Sina Company based on the allocation sequence but just as group 1 or 2. So, the clinical pharmacist remained blind regarding the treatment/placebo allocation of patients. After performing the analysis code 1 and 2 were defined by the company. So, the pharmacist, the clinical pharmacist and the emergency medicine specialist were not aware of the group allocation of the patients.

### 2.8 Statistical methods

The analysis was performed by SPSS software, version 19. Results have been reported as mean ± standard deviation for continuous variables and numbers or percentages for nominal parameters. Kolmogorov–Smirnov test was used to assess the normality of the variables distribution.

Independent sample *t*‐test and Mann–Whitney *U* test were used respectively to compare normally and non‐normally distributed variables to compare the differences between pre and postintervention. Fisher's exact test was used to compare nominal variables between the groups; *p* <.05 was considered as significant.

## 3 RESULTS

### 3.1 Baseline characteristics

**FIGURE 1:**
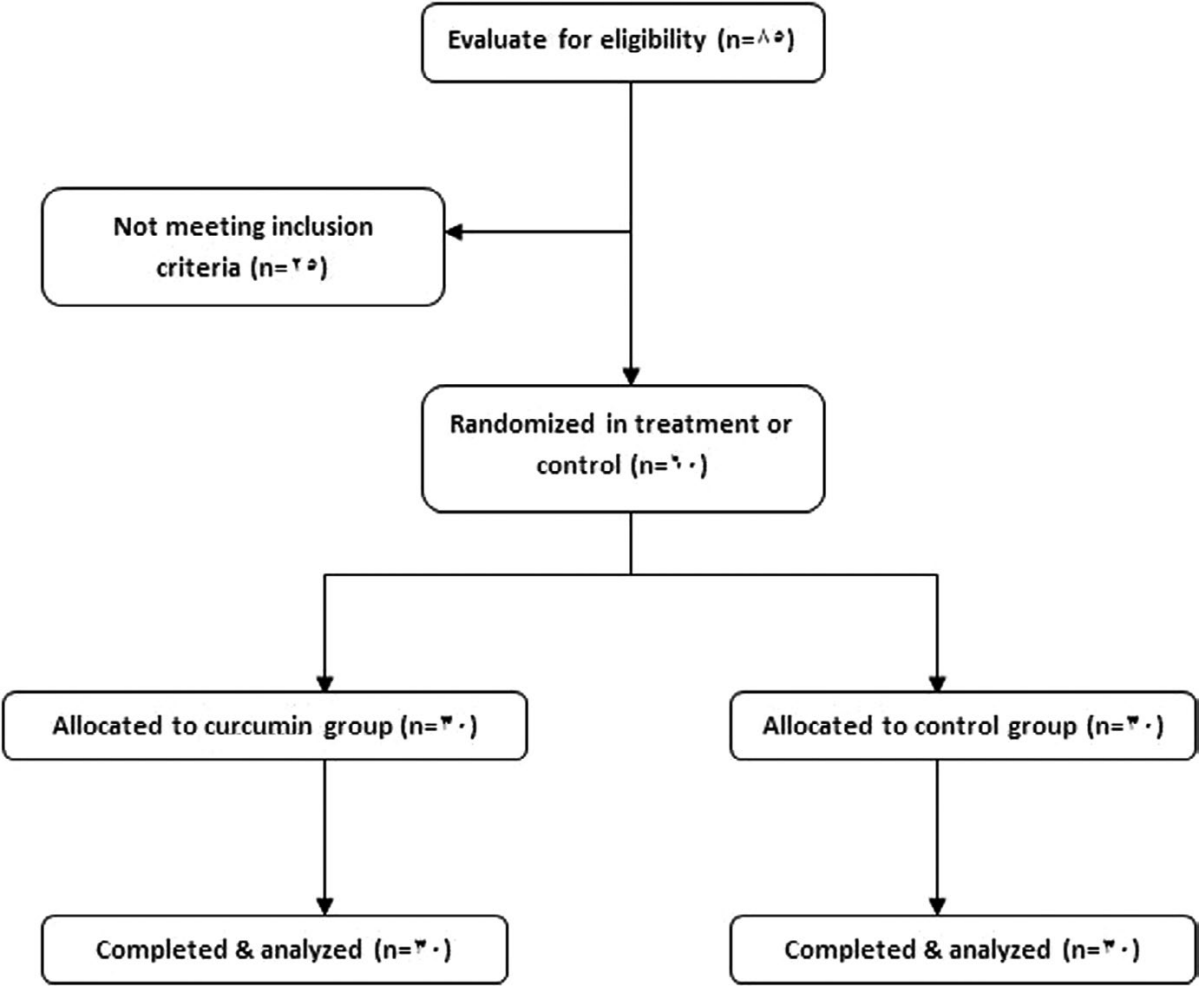
Flow diagram of the study design

Among 85 evaluated patients according to inclusion criteria, sixty patients were eligible to be enrolled in the study. Three patients were excluded during follow‐up period because of the infection exacerbation. At the end of the study, 30 patients in treatment and 27 patients in placebo group completed the trial (Figure 1). However, as our analysis method is intention to treat, we included all 60 patients to the analysis.

The average age of patients who completed the study was 43.15 ± 11.58 years, and 58.3% of them were male. Other baseline characteristics of patients are summarized in Table 1. There were no significant differences between nanocurcumin and placebo groups in these characteristics.

**TABLE 1:** Patients’ characteristics

	Nanocurcumin	Placebo	*p* value*
Gender (male/ female ratio)	20/10	15/15	0.295^a^
Age (year)	41.33 ± 12.04	44.97 ± 11	0.227^b^
Duration of symptoms’ occurrence (day)	4.63 ± 1.79	5 ± 1.76	0.427^b^
Body temperature (degree centigrade)	37.65 ± 0.76	37.63 ± 0.63	0.927^b^
Cough (%)	63.33	86.67	0.072^a^
Headache (%)	70	60	0.589^a^
Sore throat (%)	43.33	43.33	1^a^
Chills (%)	53.33	70	0.288^a^
Myalgia (%)	73.33	70	1^a^
Weakness (%)	10	30	0.104^a^
Dyspnea (%)	26.67	23.33	1^a^
Olfactory and taste disturbances (%)	60	70	0.589^a^
GI disturbances (%)	30	53.33	0.115^a^
Dermatological disturbances (%)	6.67	6.67	1^a^
Serum level of CRP (qualitative)	3 (0–3)	3(1–3)	0.707^c^
Lymphocytes count (number/µL)	2016 ± 1,294	1557 ± 1648	0.456^b^
Atrial O2 saturation (%)	94.75 ± 1.53	93.31 ± 2.57	0.064^b^

^a^ Fisher's exact test.

^b^ Independent sample *t* test.

^c^ Mann–Whitney test.

* *p* < 0.05 is considered significant.

The PCR test for COVID‐19 was performed in 6 and 5 patients in treatment and placebo groups, respectively which was positive in all of these cases (based on national guideline, this test do not perform for all suspected COVID‐19 patients in outpatient setting). Moreover, 22 & 25 patients in treatment and placebo group had some typical appearance of lung involvement based on lung CT scan.

Administration of azithromycin was the most common standard regimen in both treatment and placebo groups (25%). The other prescribed regimens are summarized in Table 2 which was not significantly different between these two groups (*p* = 0.215).

**TABLE 2:** Comparison of prescribed anti‐COVID‐19 regimens between treatment and placebo group

Anti‐COVID−19 regimen	Treatment	Placebo	*p* value^a^
HCQ (%)	0	10	0.215
Azithromycin + HCQ (%)	30	13.33	
Azithromycin + HCQ+naproxen (%)	13.33	26.67	
Azithromycin + naproxen (%)	20	20	
HCQ + naproxen (%)	10	3.33	
Naproxen (%)	0	3.33	
Azithromycin (%)	26.67	23.33	

Note

HCQ: hydroxycholorquine.

^a^ Fisher's exact test, *p* < 0.05 is considered significant.

### 3.2 Efficacy of treatment

Comparing the resolution time for various symptoms related to COVID‐19 infection between treatment and placebo group, all of them except sore throat resolved faster in treatment group. However, the difference was significant just for cough (*p* = 0.043), chill (*p* = 0.013), myalgia (*p* = 0.043) and taste and smell disturbance (*p* = 0.032) (Table 3). CRP serum level was also lower in treatment group after 2 weeks of treatment but the difference was not meaningful. However, the lymphocyte count increased more significantly in treatment group (*p* = 0.05) (Table 3).

**TABLE 3:** Comparison of symptoms resolution time and laboratory findings’ changes between treatment and placebo group

	Nanocurcumin	Placebo	*p* value*
Symptom resolution time (day)			
Fever	2.86 ± 1.65	3.6 ± 3.3	0.373^a^
Cough	4.84 ± 4.29	6.96 ± 3.87	0.043^a^ ^,^ a
Headache	3.04 ± 1.94	3.88 ± 2.06	0.211^a^
Sore throat	4 ± 2.24	3.31 ± 2.29	0.443^a^
Chills	1.93 ± 0.46	2.6 ± 0.99	0.013^a^ ^,^ a
Myalgia	3.08 ± 2.75	4.38 ± 3.01	0.043^a^ ^,^ a
Weakness	6.33 ± 3.21	7 ± 3.74	0.298^a^
Dyspnea	8.37 ± 3.92	8.62 ± 2.88	0.887^a^
Olfactory and taste disturbances	3.56 ± 2.01	5.14 ± 3.37	0.032^a^ ^,^ a
GI disturbances	5 ± 2.88	7 ± 3.72	0.164^a^
Dermatological disturbances	2	3 ± 1.41	0.5^a^
Laboratory findings’ changes			
Serum level of CRP (qualitative)	1 (0–2)	2(0–3)	0.12^b^
Lymphocytes count (number/µL)	5,440 ± 62.22	2,198 ± 948	0.05^a^ ^,^ a

^a^ Independent sample *t* test.

^b^ Mann–Whitney test.

* *p* < 0.05 is considered significant.

It is also noteworthy to mention that COVID‐19 infection deteriorated in three patients from placebo group and they were hospitalized.

### 3.3 Safety of treatment

One patient in placebo group experienced diarrhea and pruritus 2 days after inclusion to the study. Another patient from placebo group also had diarrhea at second day of treatment. However, as they received other medication including azithromycin and hydroxycholoroquine we could not definitely find out the cause of these reactions. Moreover, these symptoms could be manifestation of COVID‐19.

## 4 DISCUSSION

Present study was first randomized triple‐blind placebo‐controlled trial that assessed the efficacy and safety of nanocurcumin oral formulation in treatment of patients with diagnosis of mild to moderate COVID‐19 in outpatient setting. In this study almost all symptoms resolved faster than placebo group and the difference between two groups was significant for some important symptoms like cough, chills, myalgia and taste and smell disturbances and also lymphocytes count. Moreover, no patient in treatment group experienced deterioration of infection to the extent requiring hospitalization.

Curcumin, the bioactive ingredient of turmeric, has shown antiviral activities against several different viruses, and could be a therapeutic option for the management of COVID‐19 infection. (Babaei et al., 2020; Zahedipour et al., 2020).

In this study Sinacurcumin was used in order to improve the oral bioavalability of curcuminoids. Curcumin is a lipophilic agent and its absorption from gastrointestinal tract with normal dosage forms such as capsules and tablets is very low due to its water insolubility. SinaCurcumin is a Nanomicellar form of curcuminoids with an average size of 10 nm. Nanomicelles dissolve the active ingredient, curcuminoids, in their lipophilic part with an encapsulation efficiency of 100%. and significantly increases the water solubility. The soft gels of Sinacurcumin are completely dissolve in the acidic condition of stomach and the nanomicelles are released which are stable up to 6 hr in this conditions. This time is well enough for nanomicelles to pass the stomach and reach to the intestine, where they can absorb with different mechanisms. Our previous study has shown that bioavailability of nanomicellar curcuminoids is 59 times more than free curcuminoids. (Hatamipour et al., 2019).

Although there is no clinical trial in this field, curcumin was proposed as a possible effective measure for COVID‐19 management theoretically in some articles. Zahedipour et al. defined that curcumin could interact with various molecular targets like DNA polymerase, thioredoxin reductase, focal adhesion kinase (FAK), protein kinase (PK), tubulin, and lipoxygenase (LOX) and trigger cellular signaling pathways such as apoptosis and inflammation. Moreover, curcumin modulates intercellular signaling cascades which are crucial for effective virus replication such as attenuation of NF‐κB and PI3K/Akt signaling. It also affects cellular post‐transcriptional and post‐translational modifications. So, curcumin may have beneficial effects against COVID‐19 infection by interacting with attachment and internalization of SARS‐CoV‐2 in different organs, like liver, cardiovascular system, and kidney. It could also modulate cellular signaling pathways such as inflammation, apoptosis, RNA replication and also stimulate IFNs and other cytokines. Curcumin may also suppress pulmonary edema and fibrosis‐associated pathways in COVID‐19 infection which may be more assessable in severe COVID‐19 infection. (Zahedipour et al., 2020)

Considering blood anticoagulation properties of curcumin (by inhibiting platelet aggregation, COX pathway, and blocking of calcium signaling), as the SARS‐CoV‐2 coronavirus infection can be associated with a disseminated intravascular coagulopathy, hence curcumin can be proposed as an effective agent against this pathological condition. (Zahedipour et al., 2020)

Babaei et al. also proposed the same mechanisms for efficacy of curcumin in COVID‐19 infection. Besides, they mentioned that curcumin may be effective in management of myalgia and fatigue in COVID‐19 patients. (Babaei et al., 2020) For example in a randomized double‐blinded placebo‐controlled study curcumin (1,000 mg/d, 30 days) reduced stress and fatigue in people with occupational stress‐related anxiety and fatigue. (Pandaran Sudheeran et al., 2016) Even, curcumin inhibited sepsis‐induced muscle wasting by inhibiting catabolic response in skeletal muscle via blocking NF‐κB. (Alamdari et al., 2009) In our trial also we found that the myalgia improve significantly faster in treatment group which was in consistent with Babaei et al. hypothesis.

Furthermore, they hypothesized that, as bradykinin could trigger cough in these inflammatory diseases and also in patients with cough associated with ACE inhibitors (Hewitt et al., 2016), curcumin as an inhibitor of activated protein‐1 (AP‐1) (Singh & Aggarwal, 1995) can prevent the expression of IL‐6 induced by bradykinin in human airway smooth muscle cells and prevent cough. (Babaei et al., 2020) On the other hand, bradykinin induces cough by activation of bradykinin 2 (BK2) receptor (Al‐Shamlan & El‐Hashim, 2019), resulting in stimulation of COX and 12‐lipoxygenase (12‐LOX) metabolites release; which activate transient receptor potential (TRP) channel subfamily vanilloid member 1 (TRPV1) and subfamily A member 1 (TRPA1) channels result in an increase in both cough response and airway obstruction. (Rao, 2007) Many studies showed inhibitory effects of curcumin on 5‐LOX and COX‐2 (Valizadeh et al., 2020) which could be proposed as its mechanism in prevention of cough. (Babaei et al., 2020) Interestingly in our study the duration of cough was meaningfully shorter in curcumin group.

A randomized, double‐blind, placebo‐controlled trial by Valizadeh et al on 40 COVID‐19 patients in ARDS phase and 40 healthy controls were performed in Tabriz, Iran. They included 40 patients randomly in nanocurcumin (160 mg/d, for 14 d) or placebo group, beside betaferon 300 μg subcutaneously every other day until 5 days, bromhexine 8 mg tablets every 8 hr, and atorvastatin 40 mg daily. They found that the mRNA expression and cytokine release of IL‐1β, 6, and 18, TNF‐α were increased significantly in COVID‐19 patients compared with healthy control group. After 14 d treatment with nanocurcumin, a significant decrease in IL‐6 and 1β expression and secretion in serum and in supernatant was observed. However, IL‐18 mRNA expression and TNF‐α concentration were not influenced. (Valizadeh et al., 2020) So, above‐mentioned hypothesis about curcumin role in reduction of cytokine release in COVID‐19 patients, particularly patients with ARDS is reported in this study.

In another randomized double‐blind placebo‐controlled trial which was conducted in the same medical center, 40 mild COVID‐19 patients admitted in infectious disease ward and 40 severe COVID‐19 patients admitted to the ICU were randomly included in nanocurcumin (160 mg/d, for 21 d) or placebo group. Forty matched healthy controls were also selected. A meaningful decrease in Th17 cells and Th17 cell‐relate cytokines level were found in mild and severe COVID‐19 patients who received nanocurcumin compared to the placebo group and also in comparison with their baseline levels. It should be mentioned that these factors were significantly higher in COVID‐19 patients compared with control group. (Tahmasebi et al., 2020).

Beside above‐mentioned mechanisms for curcumin efficacy in COVID‐19, Airton Castro Rocha et al. (Airton Castro Rocha & Renato de Assis, 2020) and Babaei et al. (Babaei et al., 2020) suggested that based on available data showing that curcumin may either increase or decrease ACE in vivo activity and considering the controversy as to whether drugs acting on the angiotensin converting enzyme (ACE) pathway exacerbate the clinical appearance of patients affected by SARS‐CoV‐2, effect of curcumin on this pathway also could be a probable mechanism for curcumin efficacy in COVID‐19.

Another open label nonrandomized clinical trial was also performed by this research team on mild‐moderate hospitalized COVID‐19 patients to evaluate the efficacy of nanocurcumin. Patients who received Sinacurcumin soft gel with the same dosing as the present study, most of symptoms resolved significantly faster than control group. Moreover, SaO2 and lymphocytes count were meaningfully higher after 1 and 2 weeks of treatment with nanocurcumin, and duration of hospitalization was shorter. (SaberMoghaddam et al., 2021).

As most of these proposed effects of curcumin are related to its anti‐inflammatory, antioxidant, anticoagulant and antifibrosis properties, it seems that it may be more helpful in more severe hospitalized cases of COVID‐19 infection progressing to cytokine storm, which is somewhat shown in Valizadeh et al. and Saber‐Moghaddam et al. study. However, even in outpatient setting it was effective in reduction of symptoms durations. Moreover, the small sample size is another limitation of this study which makes the judgment difficult. It is also worth to mention that most of patients included in this study did not have COVID‐19 PCR test, as based on our national guideline it was not mandatory in outpatient setting. As COVID‐19 symptoms and CXR/lung CT view is not specific, including patients with just presumptive signs and symptoms and/or CXR/lung CT could be considered as a limitation of our study.

## 5 CONCLUSION

According to the results of this study, the nanoformulation of curcumin (40 mg/ soft gel) with dose of 80 mg twice daily could fasten the resolution time of COVID‐19 induced symptoms particularly cough, taste and smell disturbances and chills and increment of lymphocyte count in comparison with placebo and no significant adverse reaction is reported with curcumin. Clinical trials with larger sample size particularly on patients with more severe form of infection are recommended.

## CONFLICT OF INTEREST

Dr Mahmoud Reza Jaafari, one of the manuscript authors, is the founder of Exir Nano Sina Company. Other authors have nothing to declare. The study conforms to the Declaration of Helsinki, US, and/or European Medicines Agency Guidelines for human subjects.

## AUTHOR CONTRIBUTIONS

Conceptualization & Methodology: SE, MRJ, SMR, JT. Software: SE,SE. Data Analysis: SE. Investigation: RA, MDS, HR, MBR, MB. Writing – Original Draft: SS. Writing – Review & Editing: RA, MDS, HR, MBR, MB, SE, SE, MRJ, JT, SMR. Supervision: MRJ, SE.
